# Running Large-Scale Simulations on the Neurorobotics Platform to Understand Vision – The Case of Visual Crowding

**DOI:** 10.3389/fnbot.2019.00033

**Published:** 2019-05-29

**Authors:** Alban Bornet, Jacques Kaiser, Alexander Kroner, Egidio Falotico, Alessandro Ambrosano, Kepa Cantero, Michael H. Herzog, Gregory Francis

**Affiliations:** ^1^Laboratory of Psychophysics, Brain Mind Institute, Ecole Polytechnique Fédérale de Lausanne (EPFL), Lausanne, Switzerland; ^2^FZI Research Center for Information Technology, Karlsruhe, Germany; ^3^Department of Cognitive Neuroscience, Maastricht University, Maastricht, Netherlands; ^4^The BioRobotics Institute, Scuola Superiore Sant’Anna, Pontedera, Italy; ^5^Fortiss GmbH, Munich, Germany; ^6^Department of Psychological Sciences, Purdue University, West Lafayette, IN, United States

**Keywords:** visual crowding, neurorobotics, modeling, large-scale simulation, vision

## Abstract

Traditionally, human vision research has focused on specific paradigms and proposed models to explain very specific properties of visual perception. However, the complexity and scope of modern psychophysical paradigms undermine the success of this approach. For example, perception of an element strongly deteriorates when neighboring elements are presented in addition (visual crowding). As it was shown recently, the magnitude of deterioration depends not only on the directly neighboring elements but on almost all elements and their specific configuration. Hence, to fully explain human visual perception, one needs to take large parts of the visual field into account and combine all the aspects of vision that become relevant at such scale. These efforts require sophisticated and collaborative modeling. The Neurorobotics Platform (NRP) of the Human Brain Project offers a unique opportunity to connect models of all sorts of visual functions, even those developed by different research groups, into a coherently functioning system. Here, we describe how we used the NRP to connect and simulate a segmentation model, a retina model, and a saliency model to explain complex results about visual perception. The combination of models highlights the versatility of the NRP and provides novel explanations for inward-outward anisotropy in visual crowding.

## Introduction

Within the classic framework, vision starts with the analysis of basic features such as oriented edges. These basic features are then pooled along a feed-forward visual hierarchy to form more complex feature detectors until neurons respond to objects. A strength of modeling visual perception as a feed-forward process is that it breaks down the complexity of vision into mathematically treatable sub-problems. Whereas this approach has proven capable of explaining simple paradigms, it often fails when put in broader contexts (Oberfeld and Stahn, 2012; Clarke et al., 2014; Herzog et al., 2016; Overvliet and Sayim, 2016; Saarela et al., 2010). To fully understand vision, one needs to build complex models that process large parts of the visual field. At such scale, many aspects of vision potentially become relevant. For example, it is well known that spatial resolution is highest in the fovea and strongly declines toward the periphery of the visual field (Daniel and Whitteridge, 1961; Cowey and Rolls, 1974). In addition, analysis of the visual field occurs by successive eye movements, which often brings the most salient aspects of the visual image into the center of fixation (Koch and Ullman, 1985; Itti et al., 1998). Moreover, the brain is also able to covertly attend to salient parts of the visual field and detect peripheral objects, without requiring eye movements (Eriksen and Hoffman, 1972; Posner, 1980; Wright and Ward, 2008). Hence, a full model of vision needs many functions that each requires sophisticated modeling, but these many functions are not easy to achieve within one research lab. To utilize different aspects of vision in one coherent system, we need a platform where many experts in the various subfields of vision can combine their models and test them in experimental conditions.

Efforts to simulate many models for different functions of perception as a single system can encounter many challenges, including the following.

### Frameworks

Different models often come with very different computational frameworks. For example, one of the models might be a spiking neural network and another might be an algorithm involving a set of spatial convolutions. The models need a common simulation ground to talk to each other efficiently.

### Emulation

Even if models coming from different research groups are simple, producing computer code to efficiently and reliably emulate models can be a daunting task. Few labs have the expertise needed to produce (or reproduce) models that address rather different parts of the visual system.

### Analysis of the System

It is necessary, but often complicated, to determine the contribution of each model to the general output of the system. Moreover, competing models and hypotheses might be tested on the same data. To address these challenges, models should be treated as modules that can be easily removed from or added to the system. In the same vein, it is important to have a common visualization interface for the output of all simulated models.

### Synchronization

It might be difficult to synchronize all the models in a common simulation. For example, one model might be a simple feed-forward input-output transformation, and another model might be a recurrent neural network that evolves through time even for a constant stimulus. It is important to make sure that interactions between those models are consistent with their states at every time-step.

### Scalability

For many models, it is not straightforward to simulate the system efficiently and adapt the resource management to the workload of the simulation.

### Reproducibility

It is important for scientists to be able to reproduce and extend simulation results. This means not only access to model code but also the ability to reproduce stimuli. Contextual elements such as lighting, distance to the stimulus, stimulus eccentricity or even the display screen, might matter in a complex model system. The simulated environment should ensure a common set of stimuli for all scientists.

**FIGURE 1:**
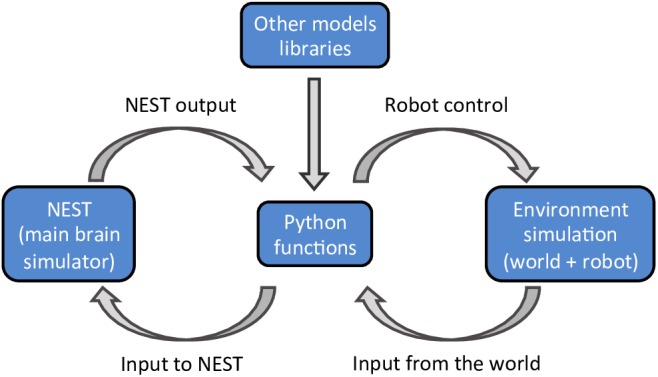
A schematic outline of the NRP components. The platform can simulate a virtual environment (right) and a NEST brain model (left). Interactions between the brain and the virtual environment are set in python functions (center). These functions also take care of models that are not simulated in NEST, importing the required libraries as python packages.

The NRP, developed within the Human Brain Project, aims to address these challenges. The NRP provides an interface to study the interactions between an agent (a virtual robot) and a virtual environment through the simulation of a brain model (Falotico et al., 2017). The platform provides tools to enable the simulation of a full experiment, from sensory processing to motor execution. The simulated brain can comprise many functions, as long as the interactions between the various functions are defined in a specified python format (Figure 1). The main brain simulator of the platform is NEST (Gewaltig and Diesmann, 2007) but the platform also supports various mathematical libraries, such as TensorFlow (Abadi et al., 2016), to implement rate based neural networks. The virtual environment, the robot, and its sensors are simulated using Gazebo (Koenig and Howard, 2004). During the simulation, the platform provides an interactive visualization of the environment and of the output of all models that constitute the brain. Importantly, the user does not have to worry about the multiple synchronizations occurring during the simulation. The platform implements a closed loop that takes care of data exchanges and synchronizations between the virtual environment, the robot, and the brain models.

Here, we show that the NRP can easily combine different visual modules, even those programmed by different research groups. We show that these combined components can explain complex observations about visual perception, taking visual crowding as an example. We made the code publicly available at https://bitbucket.org/albornet/crowding_asymmetry_nrp. In the next section, we define visual crowding and the challenges that is addresses to vision research. Then, we describe the models that are combined in our visual system and their interactions. Next, we present the results of the simulation of the visual system that we built on the NRP. Finally, we discuss the results, followed by a conclusion.

## The Case of Visual Crowding

**FIGURE 2:**
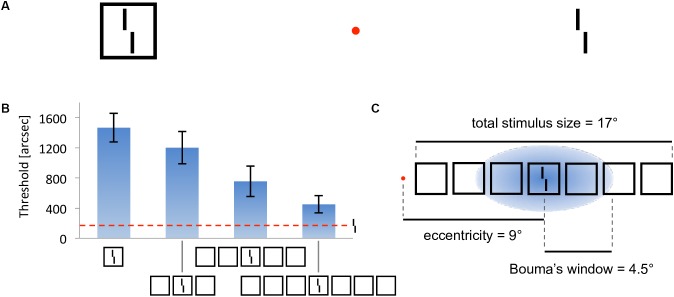
Crowding and uncrowding. Figure reproduced from Doerig et al. (2019). **(A)** Example of crowding. The task is to determine the direction of the offset of the Vernier target (tilted vertical bars), while looking at the red fixation dot. When the target is flanked by a surrounding square (left), the task is harder than when it is presented alone (right). **(B)** Uncrowding (experiment 1 of Manassi et al., 2013). Observers performed the Vernier discrimination task as presented in **(A)**, the stimuli being always displayed in the right visual field, at 9° of eccentricity. The *y*-axis shows the target-offset threshold, for which observers correctly discriminate the Vernier offset in 75% of trials (performance is good when the threshold is low). Performance for the target-only condition is shown as the dashed horizontal line. The single-square condition highlights the classic crowding effect. Importantly, adding more flanking squares improves performance gradually (Manassi et al., 2013). We call this effect uncrowding. **(C)** Performance is not determined by local interactions only. In this display, fine-grained Vernier acuity of about 200” depends on elements as far away as 8.5° from the Vernier target – a difference of two orders of magnitude, extending far beyond the hypothesized pooling region [here defined as Bouma’s window; Bouma (1970)].

**FIGURE 3:**
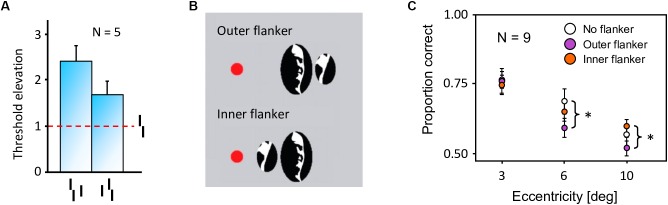
Inward-outward anisotropy in visual crowding. **(A)** Inward-outward anisotropy in a Vernier discrimination task (experiment 1b of Manassi et al., 2012). Observers performed the Vernier discrimination task as presented in Figure 2A. The stimuli were always presented in the right visual field. The *y*-axis shows the target-offset threshold elevation, which is defined as the threshold of the condition divided by the threshold of the unflanked condition. Stronger crowding is observed for an outer flanker than for an inner flanker. **(B)** Stimuli used in the crowding paradigm of experiment 5 of Farzin et al. (2009), measuring inward-outward anisotropy with Mooney faces. The red dot is the fixation point. In this paradigm, the target Mooney face is shown either in the left or the right visual hemi-field, together with either an inner flanker, an outer flanker, or with no flanker and at different eccentricities of 3°, 6°, and 10° (one block per eccentricity and per flanker configuration). Observers were asked to discriminate an upright from an inverted target Mooney face (2-AFC discrimination task). **(C)** Data from experiment 5 of Farzin et al. (2009). Note that the *y*-axis is the proportion of correct discrimination, and that a high value means a good discrimination performance. The stars indicate significant differences between conditions. The amount of inward-outward anisotropy (how much the inner-flanker condition produces better performance than the outer-flanker condition) interacts with the stimulus eccentricity.

In crowding, perception of a target strongly deteriorates when it is presented together with surrounding elements (called flankers) that share similar features with the target (Figure 2A, Bouma, 1973). As for many other phenomena, crowding was traditionally explained by local mechanisms within the framework of object recognition (Wilson, 1997; Parkes et al., 2001; Pelli, 2008; Nandy and Tjan, 2012). In this view, crowding occurs when flanking elements are pooled with target information along the processing hierarchy. Pooling can explain crowding when a few flankers are present but fails to match human behavior when more flankers are presented. For example, pooling models predict that flankers beyond the pooling region should not influence performance on the target, and that adding flankers can only increase crowding. Both predictions have been shown to be wrong. Adding flankers up to a very large distance from the target can improve performance and even fully undo crowding (Figure 2B–C; Manassi et al., 2012, 2013). Another feature of crowding that remains unexplained by pooling models is inward-outward anisotropy, which is the tendency for flankers that lie between the fixation point and the target to produce less crowding than remote flankers (Figure 3; Bouma, 1973; Petrov et al., 2007; Farzin et al., 2009; Petrov and Meleshkevich, 2011; Manassi et al., 2012).

**FIGURE 4:**
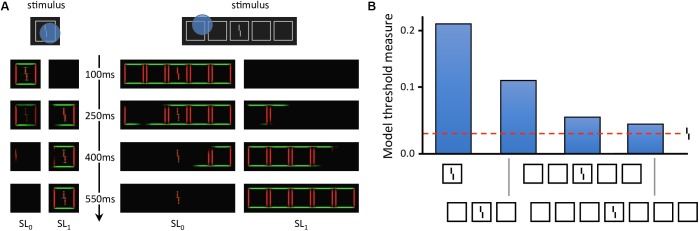
Laminart model. **(A)** Activity in the segmentation model. The intensity of each pixel corresponds to the activity of an orientation-selective neuron encoding the stimulus as a local feature detector. The color of the pixel represents the orientation of the most active neuron at that location (red: vertical, green: horizontal). Visual elements linked together by illusory contours form a potential group. The blue circles mark example locations at which the segmentation dynamics are initiated after stimulus onset. From these locations, thanks to recurrent processing, segmentation propagates along connected (illusory or real) contours, until the stimulus is represented by several distinct neural populations, called segmentation layers (two here: SL_0_ and SL_1_). Each segmentation layer represents a perceptual group. Crowding is high if other elements are grouped in the same population as the Vernier target, and low if the target is alone. On the left, the flanker is hard to segment because of its proximity to the target. Across the trials, the selection signals often overlap with the whole stimulus, considered as a single group. Therefore, the flanker interferes with the target in most trials, and crowding is high. On the right, the flankers are linked by illusory contours and form a group that spans a large surface. In this case, the selection signal can easily hit the flankers group without hitting the target. The Vernier target thus ends up alone in its layer in most trials and crowding is low. **(B)** Threshold measurement from the segmentation model’s output for all conditions of Figure 2B. The model threshold is measured by matching the output of the model to a target template over 20 segmentation trials, and then plotting the mean of the template match on a reversed axis [see Francis et al. (2017) for more details]. The segmentation model generates uncrowding and fits the behavioral data well.

Local models cannot explain these aspects of vision (Herzog and Manassi, 2015; Herzog et al., 2015; Manassi et al., 2015; Doerig et al., 2019). To fully explain crowding, one needs to take the spatial configuration of large parts of the visual field into account. Francis et al. (2017) recently explained crowding and uncrowding with a complex dynamical model that segments an input image into several distinct perceptual groups and computes illusory contours from the edges in the image. In the model, a group is defined by a set of edges that are linked by actual or illusory contours. Interference only occurs within each group, and the target is released from crowding if the flankers make a group on their own, as described in more detail below (Figure 4). However, the model does not generate inward-outward anisotropy, because it does not contain any source of asymmetry. To determine whether the grouping explanation can account for inward-outward anisotropy, we propose to incorporate the model in a more complex and realistic visual system, described in the next section.

## Materials and Methods

In this section, we describe the models that we connected, using the NRP, to explain inward-outward anisotropy in crowding. Then, we describe how the models interact with each other. The visual system is composed of the segmentation model of Francis et al. (2017), a retina model inspired by Ambrosano et al. (2016), and a saliency model, which is a simplified version of the model introduced by Kroner et al. (2019). These specific parts of human vision were chosen because the segmentation model already explains many features of visual crowding (Figure 4) and because retinal processing, as well as saliency computation, are potential sources of anisotropy for the segmentation output. Indeed, the retina model is equipped with retinal magnification and the saliency model produces a central bias. In our simulated visual system, the visual environment is first processed by the retina model and its output is sent to the segmentation model. In parallel, saliency is computed as a 2-dimensional array which corresponds to the probabilities of making an eye movement to locations in the visual field. The current simulations do not contain any eye movement, but rather use the output of the saliency model as a proxy for covert attention to determine the location where segmentation is initiated in the segmentation model. Finally, we measure crowding from the output of the segmentation model. We explain the model interactions and the crowding measurement process in more details further below.

### Cortical Model for Segmentation

The Laminart model by Cao and Grossberg (2005) is a neural network that explains a wide variety of visual properties. A critical property is the creation of illusory contours between collinear lines. Francis et al. (2017) augmented the model with a segmentation mechanism, in which elements linked by contours (illusory or real) are grouped together by dedicated neural populations. The goal was to provide a two-stage model of crowding, with a strong grouping component: stimuli are first segmented into different groups and, subsequently, elements within a group interfere. After dynamical processing, different groups are represented by distinct neural populations. Crowding is determined by matching the model’s output to a target template. Importantly, crowding is weak when the target is alone in its group (i.e., when the population representing the target does not also represent other elements) and strong otherwise.

The segmentation process is triggered by local selection signals that spread along connected contours (Figure 4). The location of the selection signals determines the output of the segmentation process. Uncrowding occurs when a selection signal touches a group of flankers without touching the target. In the original version of the model, the location of each selection signal followed a spatial distribution tuned to maximize successful segmentation of the target from the flanker in the crowding paradigm. This assumption follows the idea that, in psychophysical paradigms, an observer does the best job possible to succeed in the task. Here, we try a different approach by using the output of the saliency model to bias the location of the selection signal toward interesting regions of the visual field, as described further below.

### Retina Model

Previous work has integrated a retina model as part of a neurorobotic experiment in the NRP (Ambrosano et al., 2016) by using the COREM (Computational Retina Modeling framework; Martínez-Cañada et al., 2015, 2016). COREM is a set of building blocks that are often used to describe the behavior of the retina at different levels of detail. The system includes a variety of retina microcircuits, such as spatial integration filters, temporal linear filters, and static non-linearities. The retina model that is adopted for this work is an adaptation of a model of the X cells in the cat retina as described by Wohrer and Kornprobst (2009). We also use the COREM framework to simulate the retina model in the NRP. The model uses feedback loops between retinal layers to control contrast gain (Shapley and Victor, 1978). The X cells are chosen in this work because of their tonic and fine-grained response, as our paradigm involves highly detailed stimuli.

**FIGURE 5:**
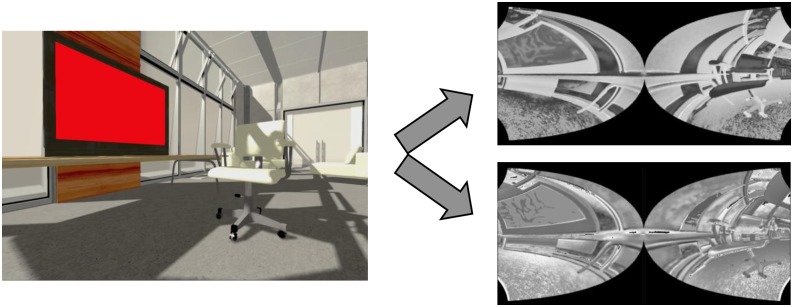
Retina model. Left: input example. Right: associated output of the retina model (OFF-centered ganglion cells on top and ON-centered ganglion cells below). We generated these input and output images by simulating the retina model on the NRP. The ON- and OFF-centered ganglion cells react to bright and dark regions of the image, respectively, and are more active around regions of high contrast. The output images look distorted, because fewer retinal ganglion cells, whose output is represented by one pixel for each cell, encode the same portion of the visual field as the eccentricity grows. For example, the left side of the TV screen looks smaller than its right side, closer to the fovea. Note that the image on the left has been rendered by the NRP and that the real input of the retina model is not rendered. For example, the shadows are not fed to the retina model, which does not impact our experimental setup because no shadows are involved in the crowding paradigms we reproduce.

In addition, we include space variant Gaussian filters provided by COREM that mimic retinal magnification. Along the retinal layers, visual information is pooled with less spatial precision in the periphery than in foveal locations because the Gaussian integration filters are broader with eccentricity. Finally, the output of the retina, i.e., the activity array of the ON- and OFF-centered ganglion cells, is distorted by a log-polar transform to mimic the magnification that results from the mapping of the retina neurons to the visual cortex. An example of the model’s output is shown in Figure 5.

### Saliency Model

Computational models of saliency aim to identify image regions that attract human eye movements when viewing complex natural scenes. The contribution of stimulus features to the allocation of overt attention can then best be captured in a task-free experimental scenario. As a model of saliency computation, we used a deep convolutional neural network, simulated in TensorFlow (Abadi et al., 2016), that automatically learns useful image representations to accurately predict empirical fixation density maps. Compared to early approaches based on biologically motivated feature channels, such as color, intensity, and orientation (Itti et al., 1998), the architecture extracts information at increasingly complex levels along its hierarchy.

**FIGURE 6:**
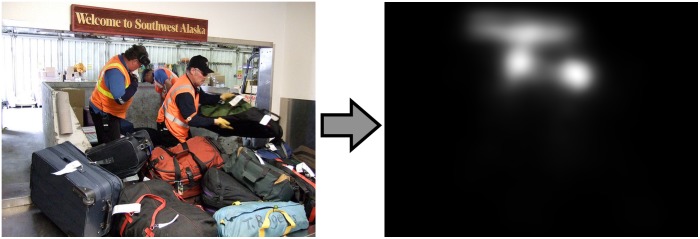
Saliency model. Left: input example that the saliency model can process. Right: corresponding saliency probability distribution that the model produces after training. Here, the most salient regions are the faces and the sign.

The model is an encoder-decoder network that learned a non-linear mapping from raw images to topographic fixation maps. It constitutes a simplified version of the model introduced by Kroner et al. (2019), pruning the contextual layers to achieve computationally more efficient image processing. The VGG16 architecture (Simonyan and Zisserman, 2014), pre-trained on a visual classification task, serves as the model backbone to detect high-level features in the input space. Activation maps from the final convolutional encoding layer are then forwarded to the decoder, which restores the input resolution by applying bilinear up-sampling followed by a 3 × 3 convolution repeatedly. The task of saliency prediction is defined in a probabilistic framework and therefore aims to minimize the statistical distance between the estimated distribution and the ground truth. The model we used in this work was trained on the large-scale SALICON data set (Jiang et al., 2015), used as a proxy for eye tracking data. After training, the model produces a saliency map for any input image, such as in Figure 6. In our visual system, the saliency model output determines where the segmentation model selects objects of interest. The local selection signals that trigger segmentation in the model follow the saliency output as a probability density distribution. Although the saliency network models the empirical distribution of overt attention across images, we use it as a proxy of covert attention to select interesting objects from the background.

#### Virtual Experiment and Model Interactions

**FIGURE 7:**
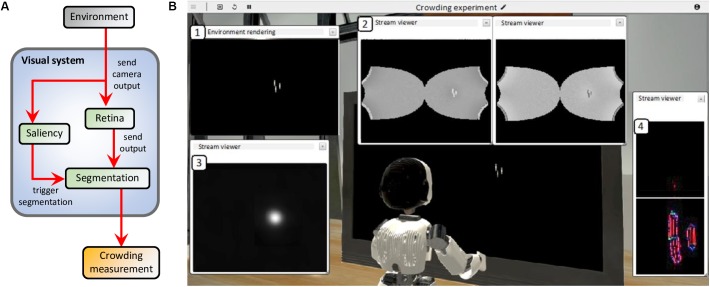
**(A)** Model interactions in the visual system (blue box) of the robot. The camera of the right eye of the robot processes the visual environment (gray box) and sends a gray-scale input image to both the retina and the saliency models. The retina model sends its output, i.e., the contrast-related activity of ON- and OFF-centered ganglion cells, to the input layer of the segmentation model. The saliency model delivers its output to the segmentation model as a 2-dimensional probability density distribution that determines where each selection signal (such as the blue circle in Figure 4) starts the segmentation dynamics, whenever the visual stimulus appears to the robot’s eyes. Finally, a threshold measurement (yellow box) is computed from the segmentation model’s output. Since neither the robot nor the robot’s eyes move, there is no arrow going from the visual system to the environment. **(B)** Example of the result of the simulation of the visual system for one segmentation trial. In this example, the environment of the robot reproduces one of the conditions of the paradigm that measures inward-outward anisotropy in visual crowding in Manassi et al. (2012; see Figure 3A). All displayed windows are interactive visualizations of the output of the models that constitute the visual system (see **A**). They can be displayed while the simulation is running. (1) Output of the camera of the right eye of the robot, which is fed to the retina and the saliency models. (2) Output of the retina model (ON- and OFF-centered ganglion cells, respectively on the right and on the left). (3) Output of the saliency model. The visual stimulus is very salient (white spot). (4) Output of the segmentation model. Each slot of the segmentation model’s output corresponds to a different segmentation layer (as in Figure 4A, except SL_0_ and SL_1_ are above and below here). The intensity of each pixel corresponds to the activity of an orientation-selective neuron encoding the stimulus as a local feature detector. The color of the pixel represents the orientation of the most active neuron at that location (red: vertical, green: horizontal, blue: diagonal, and turquoise or purple: intermediate orientations). The output associated to the stimulus is not a straight vertical line, as in Figure 4A, because the input of the segmentation model is distorted by the retina model. Here, the segmentation has not been successful, because the target and the flankers end up in the same segmentation layer. This means that at stimulus onset, the segmentation signal drawn from the saliency distribution overlapped with both the target and the flanker, spreading the segmentation to the whole stimulus.

The virtual environment reproduces the conditions of two experiments that measure inward-outward anisotropy in visual crowding (see Figure 3): experiment 1b of Manassi et al. (2012) and experiment 5 of Farzin et al. (2009). A screen displays the visual stimulus (flankers and target) to the eyes of an iCub robot at a specific distance and a specific eccentricity, depending on the conditions of the simulated experiment. In all simulated conditions, the task of the robot is to give a measure of crowding associated to the stimulus, by trying to segment the flanker from the target over many trials. For each trial, the stimulus appears in the periphery of the right visual field of the robot, while the integrated camera of the right eye of the robot constantly records its visual environment and sends its output to the visual system. To process the visual stimulus, the models of the visual system are connected to each other according to the scheme in Figure 7A.

Figure 7B shows the result of an example trial simulated with the NRP and highlights the output of all models of the visual system. When the visual stimulus (the target with either an inner flanker, an outer flanker, or unflanked) appears on the screen, the camera of the robot sends its output to the retina model whose output is delivered to the segmentation model. Because of the magnification applied by the retina model, the segmentation model represents elements in the visual field with less precision if they appear in the periphery than if they appear near the fovea. At the same time, the saliency model is also fed with the output of the camera. The saliency model is not fed with the output of the retina model because it has been trained on undistorted images. In the simulation, the output of the saliency model corresponds to a probability density distribution of the selection signals that are sent to the segmentation model (see blue circle in Figure 4). After stimulus onset, a selection signal, whose location is sampled from the saliency map intensity, starts the segmentation dynamics of the segmentation model. The selection signal is sent to locations near the visual stimulus, because it is very salient. After some processing time, the segmentation stabilizes (groups are formed in the segmentation layers). The location of the selection signal drives the output of the segmentation. If it overlaps with both the target and the flanker, the segmentation is unsuccessful because the flanker and the target interact. If not, the segmentation is successful because the target ends up alone in its segmentation layer. When the target disappears, the activity of the segmentation model is reset by an overall inhibition signal, and the loop starts over.

**FIGURE 8:**
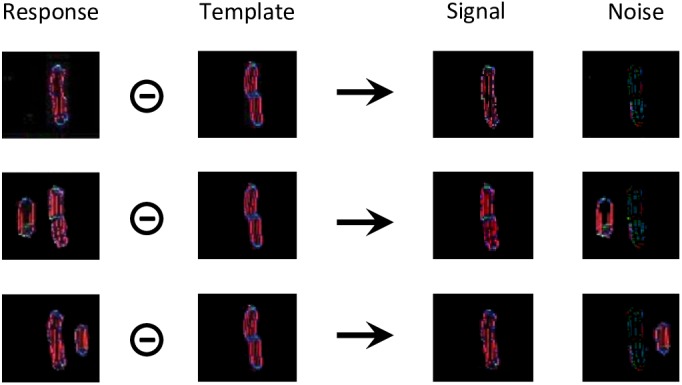
Threshold computation, taking as an example the output generated by the segmentation model for all stimuli of experiment 1b of Manassi et al. (2012; see Figure 3A). The output of the segmentation model for these stimuli do not look like straight lines, because the input is distorted by the retina model. For all these conditions, the target corresponds to the shape in the template array. The template was built by presenting the target alone to the visual system and taking the mean of the segmentation model’s output over several time-steps. The circled minus sign represents the following computation. After taking the mean of the arrays over all orientations, any pixel from the response array is multiplied by the value of the same pixel of the template array to obtain the value of the same pixel in the signal array, and by 1 min the value of the same pixel of the template array to obtain the value of the same pixel in the noise array. In other words, the pixels that match the template are assigned to the signal, and the ones that do not correspond to it are assigned to noise. Then, the threshold is computed as a measure of interference between the signal and the noise arrays, according to equation (1).

For each condition of experiment 1b of Manassi et al. (2012) and experiment 5 of Farzin et al. (2009; Figure 3), we simulate the visual system of the robot for 20 trials. For each trial, we record a threshold measurement, based on the output of the segmentation model. First, we compare the output array to a target template to separate it into a signal and a noise array (Figure 8). The target template is the mean of the segmentation model’s output over several time-steps that is generated when the target is presented alone.

Those signal and noise arrays are then used to measure the match M between the output of the segmentation model and the target template, according to equation (1).

(1) $$M=\sum_{i,j}(s_{\mathrm{ij}}-\sum_{k,l}n_{\mathrm{kl}}\cdot I_{0}\cdot e^{-\frac{\sqrt{{\left(i-k\right)}^{2}+{\left(j-l\right)}^{2}}}{\sigma }})$$

The intensity of pixel (i, j) of the signal array is denoted by s_ij_ and the intensity of pixel (k, l) of the noise array by n_kl_. The weight of interference between those two pixels decreases exponentially with the distance between them. I_0_ is the strength of interaction and sigma is the rate of exponential decrease. I_0_ is set to 10^-3^, a value that was determined to generate sufficient interaction between the target and the flanker, without killing the signal completely. Sigma is set to 30 pixels, a value that was determined to follow approximately the pooling range defined by Bouma’s window (Bouma, 1970). Given this fixed value, the pooling range increases with eccentricity in the image space. The more flanker elements, in addition to the target, that are in the segmentation layer, the smaller the match. Note that even for a fully successful segmentation trial, when the target ends up completely alone in one of the segmentation layers, the match is not perfect, because the representation of the target has intrinsic noise and dynamics and thus does not perfectly match the template (Figure 8, first row). Also note that a small target generates less signal, and thus a weaker match, than a larger version of the same target. Difficulty of judging Vernier direction is usually measured by identifying the threshold separation needed for an observer to be 75% correct. In the model, we suppose that the threshold is a negative linear function of the match value (the higher the match, the lower the threshold), exactly as in Francis et al. (2017).

Finally, for each condition, we take the mean of the thresholds (T_i_) across the trials and divide this value by the mean thresholds of the unflanked condition, where only the target is presented to the robot. We define this final number as the model measurement of the threshold elevation of the flanking configuration [see equation (2)].

(2) $$E_{i}=\frac{\frac{1}{N}\sum_{n=1}^{N}T_{i}(n)}{\frac{1}{N}\sum_{n=1}^{N}T_{u}(n)}$$

Where E_i_ is the threshold elevation of condition i, N is the number of trials, T_i_(n) is the threshold measurement associated to the segmented output of trial n for condition i, and T_u_(n) is the threshold measurement associated to the segmented output of trial n for the unflanked condition.

## Results

### Vernier Discrimination Task

**FIGURE 9:**
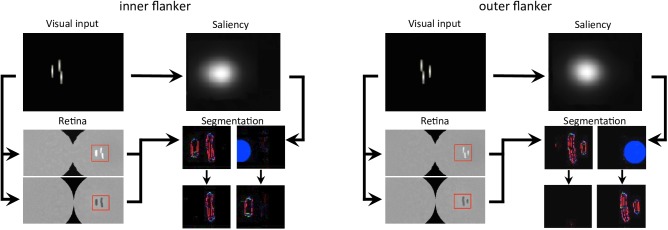
Output of all models, for both flanked conditions of experiment 1b of Manassi et al. (2012; see Figure 3A). The arrows represent the interactions that are described in Figure 7A. In the visual input and the saliency windows, the position of the fixation point corresponds to the center of the leftmost column. The retina window shows the output of the ON- and OFF-centered ganglion cells at the top and the bottom, respectively. The red rectangle highlights the portion of the ganglion cells output that is fed to the segmentation model, to gain computation time. The segmentation window shows the initial state of the model output in the first row, with an example of a selection signal occurrence, drawn from the saliency distribution (blue circle), and the resulting output of the model in the second row, after the segmentation dynamics have stabilized. Each column of the segmentation window corresponds to one segmentation layer, as in Figure 4. Here, the inner flanker condition led to a successful segmentation trial, and the outer flanker condition led to a failed segmentation trial.

**FIGURE 10:**
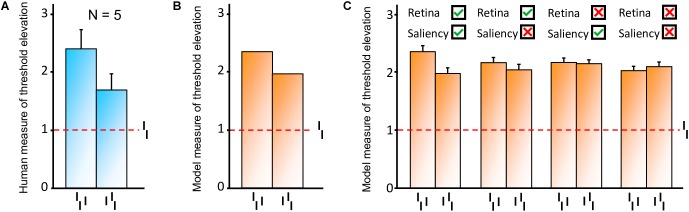
Model results, reproducing the conditions of inward-outward anisotropy in experiment 1b of Manassi et al. (2012; see Figure 3A). In each bar graph, the red dashed line shows the threshold for the unflanked condition (Vernier target alone). To compare the model with the data, we measured threshold elevation defined as the threshold of a condition divided by the threshold of the unflanked condition (see Methods section). **(A)** Behavioral data from experiment 1b in Manassi et al. (2012). **(B)** Simulation results obtained with the full visual system (retina, saliency, and segmentation). Contrary to the human data, we cannot compute error bars across observers because only a single set of model parameters is used in the simulations. The model fits the human data well, producing a similar anisotropy. **(C)** Comparison of the simulation results with and without the activation of the different modules of the visual system. Error bars were computed by simulating the system over 10 sessions (20 trials per session for each condition). When the retina model is inactive, the camera of the robot sends its signal directly to the segmentation model. When the saliency model is inactive, the selection signals are sent as they were in the original version of the segmentation model, i.e., sampling their location according a two-dimensional Gaussian distribution centered on the location that maximizes segmentation success. The best fit comes from the full visual system, and a bigger threshold elevation for the outer flanker condition, compared to the inner flanker condition, is generated only when the retina model is active.

**FIGURE 11:**
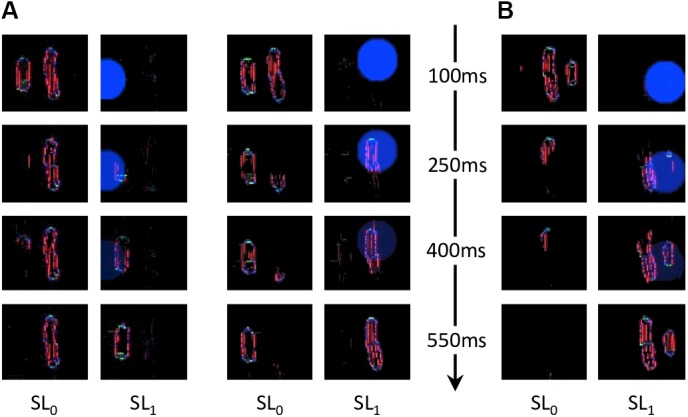
Characteristic examples of segmentation processes for both conditions of experiment 1b of Manassi et al. (2012). Every row corresponds to the segmentation model’s output at a certain time after stimulus onset, indicated by the arrowed axis. Each pair of columns corresponds to the output of a simulation, and the content of each segmentation layer is indicated by SL_0_ and SL_1_ (as in Figure 4A). **(A)** Two examples of successful segmentation trials for the inner flanker condition. **(B)** Example of a failed segmentation trial for the outer flanker condition. The probability of successfully segmenting the flanker from the target is higher in the outer flanker condition than in the inner flanker condition. The inner flanker is better represented by the retina output than the outer one, because it is presented closer to the fovea. The inner flanker appears bigger and further from the target. The resulting threshold elevation for the inner flanker condition is thus lower than for the outer flanker, corroborating the inward-outward anisotropy measured in experiment 1b of Manassi et al. (2012). Both conditions often lead to unsuccessful segmentation because the flankers are quite close to the target, given the eccentricity of the stimulus, and because the saliency model’s output computes the whole stimulus as only one object (see Figure 9). Thresholds for both flanked conditions are hence substantially larger than for the unflanked condition.

First, we reproduced the crowding paradigm of experiment 1b of Manassi et al. (2012; see Figure 3A). This experiment measured inward-outward anisotropy in a Vernier discrimination task. In the simulation, we showed a Vernier target at a fixed eccentricity of 3.89° from the fovea in the right visual field of the robot. The target was either flanked by a short bar on the left side, on the right side, or not flanked at all. Representative outputs of the retina model, the saliency model, and the segmentation model for both flanked conditions are presented in Figure 9. The threshold measurements for all conditions, coming from the NRP simulation as well as from the behavioral data, are shown in Figure 10A–B. To investigate the role of each model in the general output of the system, we de-activated the different modules of the visual system and measured the corresponding model output thresholds (Figure 10C). Crucially, the simulation of the full visual system (retina, saliency and segmentation models) produces the best fit of the data (i.e., a larger threshold when the target was flanked by an outer bar than when flanked by an inner bar). De-activating only the saliency model in the visual system also generated the same kind of asymmetry as in the data, but to a smaller extent, suggesting that the retina is the main source of asymmetry in this paradigm. Indeed, an inner flanker is better represented by the retina model than an outer flanker, because it appears at a smaller eccentricity. When the flanker is presented on the foveal side, its representation is bigger and appears further from the target, and the segmentation model is more prone to segregate it from the target. This small but crucial difference between both flankers is illustrated in Figure 11.

### Mooney Face Discrimination Task

**FIGURE 12:**
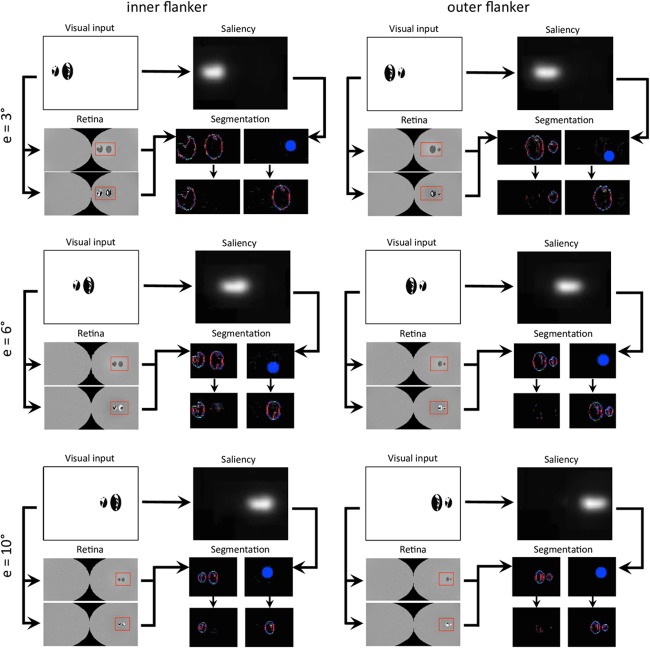
Output of all models and for all conditions of experiment 5 of Farzin et al. (2009; see Figure 3B–C). The description of the windows for each condition is the same as in Figure 9. Each row of conditions displays the output of the visual system for a specific eccentricity. Note that the visual stimulus appears smaller in the retina model’s output (and hence in the segmentation model’s output) as the eccentricity grows. To highlight how different it is to segment the flanker from the target for various eccentricities, the output of the retina model as well as the segmentation model have the same scale across the conditions (e.g., the selection signal always has the same size).

**FIGURE 13:**
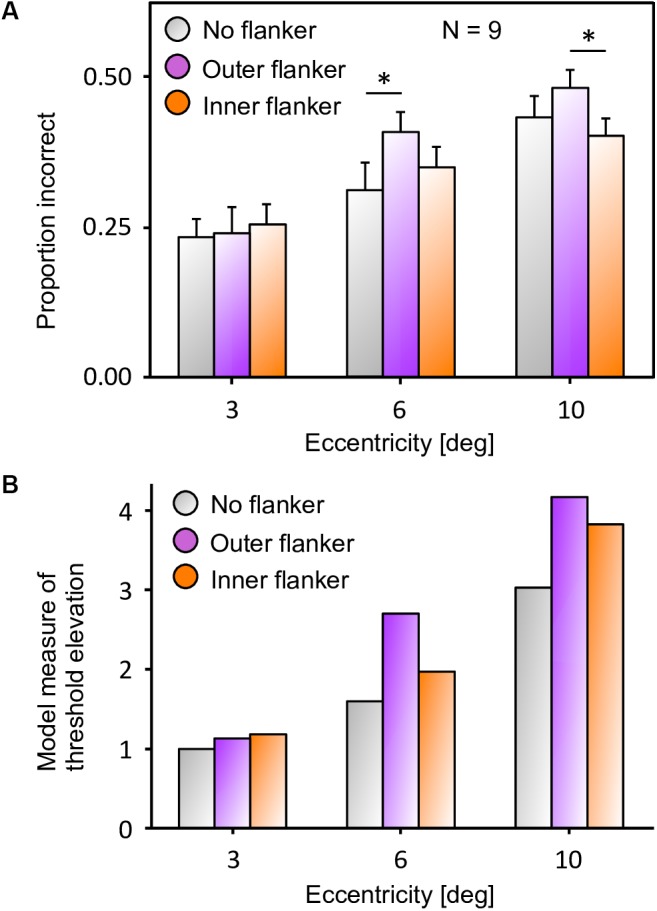
**(a)** Data from experiment 5 of Farzin et al. (2009), that measures inward-outward anisotropy in visual crowding with Mooney faces. The figure has been re-drawn from Figure 3C as bars that report the proportion of incorrect trials, to compare to the model results. Here, a value closer to the top corresponds to a bad performance, like in a threshold measure. The amount of inward-outward anisotropy (how much the inner flanker condition differs from the outer flanker condition) varies with eccentricity. The stars indicate significant differences between conditions. **(B)** Threshold elevation measurement obtained with the simulation of the full visual system (retina, saliency, and segmentation), reproducing all conditions of the original experiment on the NRP. To compute the threshold elevation for each condition, we divided each threshold by the threshold of the unflanked condition at 3° of eccentricity (the lowest threshold value). The model threshold measurements highlight the same interaction as in the data, between the eccentricity and the amount of inward-outward anisotropy. Ranking the model threshold elevation measurements from the lowest to the highest value almost perfectly matches the data, ranking from the highest to the lowest performance. The only difference is that the threshold elevations that the model produces for the unflanked and the inner flanker condition are swapped at 10° of eccentricity (the model predicts that the unflanked condition is always better than both flanked conditions at the same eccentricity). In terms of quantitative differences, the model produces more inward-outward anisotropy for 6° than for 10° of eccentricity, which does not fit the data (the data shows a significant difference between the inner and the outer flanker condition for 10° but not for 6° of eccentricity).

**FIGURE 14:**
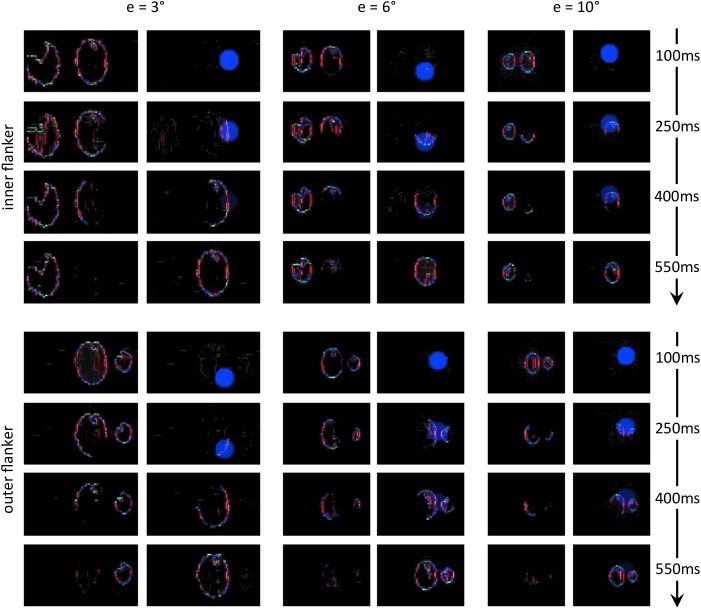
Characteristic examples of segmentation processes for all conditions of experiment 5 of Farzin et al. (2009; see Figure 3B–C). The output of the segmentation model has the same scale across the conditions (e.g., the selection signals always have the same size). For both conditions at 3° of eccentricity, the retina model represents the stimulus with such details, that the task is easy even for an outer flanker, resulting in a high rate of segmentation success across the trials, and no substantial difference of threshold between those conditions. For larger eccentricities, the whole stimulus representation has a lower resolution. The target appears smaller, and the flankers appear closer to the target. Many trials thus lead to unsuccessful segmentation, because the selection signals have only a very small region to select only one of the objects. At the same time, the inner flanker is always better represented than the outer flanker. The model thus generates differences of thresholds between the inner-flanker and the outer-flanker conditions.

Next, we reproduced the crowding paradigm of experiment 5 of Farzin et al. (2009; see Figure 3B–C). This experiment measured inward-outward anisotropy using Mooney faces. In this paradigm, the target Mooney face is shown either in the left or the right visual hemi-field, together with either an inner flanker, an outer flanker, or with no flanker and at different eccentricities of 3°, 6°, and 10° (one block per eccentricity and per flanker configuration). Observers were asked to discriminate an upright from an inverted target Mooney face (2-AFC discrimination task). We performed the same model measurements as in the previous simulations. We ran the visual system and collected threshold elevation results for all different eccentricities of the original experiment; presenting the Mooney face target together with either an inner or an outer flanker. The outputs of the retina model, of the saliency model, and of the segmentation model in response to all conditions are presented in Figure 12. The threshold measurements, coming from the NRP simulation as well as from the behavioral data, are shown in Figure 13. The simulation generates the same interaction between the eccentricity and the amount of inward-outward anisotropy that is found in the empirical data. A substantial difference of threshold elevation between the inner flanker and the outer flanker conditions is measured only for big eccentricities (6° and 10°). The reason is that for a small eccentricity (3°), the representation of the target generated by the retina model is so big that the segmentation is successful in almost every trial. For an inner flanker, the region to select only one of the objects is very large, and the selection signals thus have a very low probability of hitting both the target and the flanker at the same time. For an outer flanker, even if the flanker region gets substantially smaller, the target region is still very big, and most of the selection signals fall on the target, also leading to a very high segmentation success rate. In other words, the task is too easy to highlight any difference between the inner and the outer flanker conditions. For larger eccentricities, the size of the retina output associated with the stimulus becomes smaller, which makes the task more difficult. Over the trials, many selection signals can be unsuccessful (fall on both the target and the flanker) for both inner and outer flanker conditions, highlighting substantial differences in their threshold measurements. Those critical differences between the conditions are illustrated in Figure 14.

## Discussion

Using the NRP, we simulated a complex visual system composed of several models coming from different research labs. The platform provides satisfactory answers to many of the challenges described in the Introduction. Here, we summarize these issues and briefly explain how the NRP addresses them.

### Frameworks

Even if the models that we use have different computational frameworks, the platform allows us to easily integrate them into a common visual system, define their interactions, and simulate them with a minimal amount of code. For example, the segmentation and the saliency models use NEST and TensorFlow, respectively, which the platform supports.

### Emulation

The collaborative aspect of the platform made it possible to quickly integrate the retina model to the simulation. The retina-modeling framework was already incorporated to the platform by other users (Ambrosano et al., 2016), together with some documentation and examples.

### Analysis of the System

The NRP allows researchers to de-activate models, simply by commenting out a single line in the setup file of the virtual experiment. This is a powerful tool to investigate how each model contributes to the general output of the system (see Figure 11C), or to test competing hypotheses (e.g., compare how two competing models for the same function of vision fit some data).

### Synchronization

The platform takes care of the synchronization between the simulated models. In our visual system, the segmentation model is a recurrent network and the saliency model is a feed-forward input-output transform and the NRP ensures that their respective inputs are always consistent. The models are first run in parallel for a short amount of time. Then the platform collects data from the simulation and computes the relevant inputs for the next simulation step.

### Scalability

However, some challenges were handled with less success. Simulating the whole visual system with the required input resolution required very long computational times (2 weeks to simulate all conditions). The platform is currently used online with servers that have rather limited resources. The platform is in development and will soon support high-performance computing.

### Reproducibility

Because of the computational limitations, we could not reach the resolution that was required to identify the high-level features of some stimuli (e.g., “face-ness” of the Mooney faces). It would be interesting to check if the “face-ness” of the Mooney faces drastically changes the output of the saliency model and if the model threshold results substantially change.

Ultimately, simulating the visual system on the NRP allowed us to enhance understanding about visual crowding. We could show that the segmentation model that explains crowding and uncrowding (Manassi et al., 2012, 2013; Francis et al., 2017) is able to explain inward-outward anisotropy as well, if it is connected to a retina model. Traditional explanations of crowding (e.g., pooling models) combined with retinal and cortical magnification would predict that an outer flanker produces less crowding than an inner flanker. The representation of an outer flanker in the visual cortex would appear smaller than the one of an inner flanker, thus causing less interaction with the target through pooling, whose range is expressed in cortical distance. Here, on the contrary, simulating the segmentation model of Francis et al. (2017) in a complex visual system, the prediction is exactly the opposite, thereby matching the data. Indeed, it becomes harder for the visual system to segment the flanker from the target, if the representation of the flanker is small. In other words, the visual system is more likely to treat the flanker and the target as a single object (or group). The grouping hypothesis of Francis et al. (2017) can thus explain uncrowding as well as inward-outward anisotropy. This gives more evidence to the idea that grouping is a central function of human vision (Manassi et al., 2012; Chaney et al., 2014; Harrison and Bex, 2016; Doerig et al., 2019).

The full model simulated with the NRP makes the prediction that inward-outward anisotropy can be observed only for a fixed range of eccentricities. If the eccentricity is too small (e.g., 3° for the paradigm of Farzin et al. (2009); see Figure 13, 14), no difference can be observed between the inner flanker and outer flanker conditions because the segmentation is almost always successful in both cases. Indeed, the retinal output related to the visual stimulus is substantially larger than the selection signals, and the probability that the signal covers both the target and the flanker is very low. If the eccentricity is too large (i.e., even bigger eccentricities than in Figure 13, 14, e.g., 13°, 16°, or 20°), an inner or an outer flanker becomes indistinguishable from the target, because the stimulus is represented as a tiny spot by the retina. The selection signal of the segmentation model would always cover the whole stimulus, segmenting the target and the flanker as a single group, thereby making no difference between an inner and an outer flanker. In Figure 13, the model produces a stronger inward-outward anisotropy for 6° than for 10° of eccentricity, which does not fit the human data. We attribute this discrepancy to a sub-optimal choice of the size of the selection signals in the segmentation model (the radius of the blue circles, e.g., in Figure 14). As said above, the radius of the selection signals directly affects the range of eccentricity at which inward-outward anisotropy is observed. If the signals were smaller, the eccentricity at which inward-outward anisotropy is maximal would be larger and vice versa. In general, this tells us that a more sophisticated mechanism should be used to trigger segmentation events. For example, at stimulus onset, the saliency output could instantiate a soft neural competition to determine the location and the size of the selection signal. A threshold, put on the time derivative of all pixel intensities of the saliency output, could even be used to determine when and where to trigger such a competition.

**FIGURE 15:**
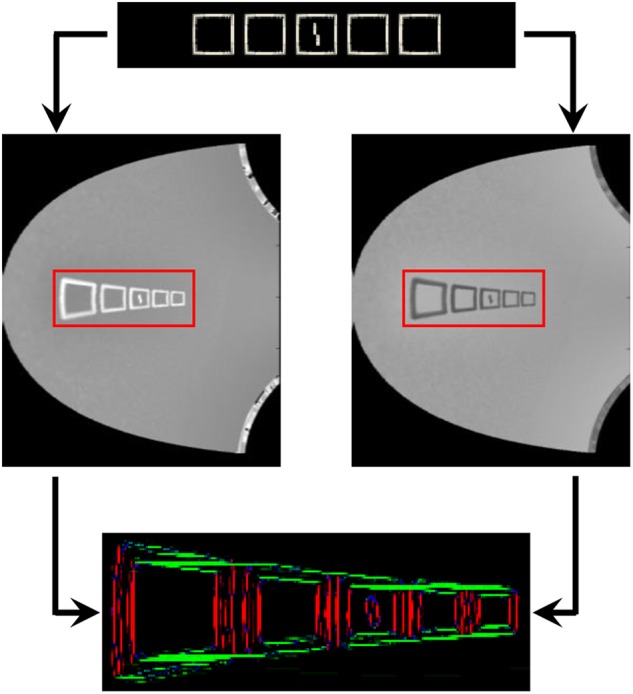
Formation of illusory contours in the full visual system for the 5-square-flankers condition of Figure 4A. The image on the top is the visual input to the visual system, both images in the middle are the output of the ON-centered (left) and OFF-centered (right) ganglion cells of the retina model (only the right visual field), and the image on the bottom is the output of the segmentation model. Illusory contours are formed between almost all squares, but they sometimes come from the alignment of the very top of one square with the inner part of the top of the other square.

Furthermore, it would be interesting to test how inward-outward anisotropy interacts with uncrowding. A new interesting paradigm would be to continue the experiment 1b of Manassi et al. (2012) with different numbers of short flanking bars. Previously, it has been shown that crowding weakens when adding more bars on both sides of the target, if they are aligned with each other (experiment 1a of Manassi et al. (2012)). To simulate such paradigms, we need to investigate whether our model of the visual system allows the creation of illusory contours between aligned flankers, such as between the squares of Figure 4A, to produce uncrowding. We expect that the distortion due to the retina model impairs the formation of illusory contours between aligned edges, because the segmentation model assumes that spatial pixels correspond to retinal pixels (see Francis et al. (2017) for the exact mechanism). We reproduced the 5-square-flankers condition of Figure 4A in the NRP and we simulated the model visual system (Figure 15). The segmentation model still generates illusory contours but to a lesser extent. We suspect that the mechanisms need not be changed but the way an aligned neighbor is encoded in the model should be redefined. This simulation highlights how challenging it is to merge different models. The NRP forces us to recognize a challenge in integrating the retina and the segmentation model. Future work is thus needed in order to simulate this kind of paradigm properly.

## Conclusion

Breaking down the complexity of vision into simple mechanisms fails when the simple mechanisms are put in broader contexts. To fully understand human vision, one needs to build complex systems that process large parts of the visual field and combine many aspects of vision that all require sophisticated modeling. Using the NRP, we could start to simulate such a system by connecting a segmentation model, a saliency model, and a retina model, thereby providing explanations for complex results in visual crowding, such as inward-outward anisotropy. Crucially, the explanation is in line with the grouping hypothesis of Francis et al. (2017) and predicts how much inward-outward anisotropy would be measured at bigger eccentricities. This early use of the NRP suggests that it provides a solution to some of the challenges that come with simulating big connected systems. We believe the system will prove useful beyond the specific models utilized here; and that it will provide a common platform for general purpose modeling of perception, cognition, and neuroscience.

## Data Availability

No datasets were generated or analyzed for this study.

## Author Contributions

AB, JK, AK, and AA substantially contributed to conducting the underlying research. AB, AK, and AA provided the models descriptions to the manuscript writing process. KC provided the description of the Neurorobotics Platform to the manuscript writing process. AB wrote most of the manuscript and put all parts together. GF, MH, EF, JK, and AK gave substantial feedbacks to the writing process.

## Conflict of Interest Statement

KC was employed by the company Fortiss GmbH. Fortiss GmbH is a public research institute financed by the Bavarian region. It is the principal developer of the NRP. The remaining authors declare that the research was conducted in the absence of any commercial or financial relationships that could be construed as a potential conflict of interest.
